# Analysis of Green House Home Performance Across Domains of the CMS Nursing Home Five-Star Quality Rating System

**DOI:** 10.1093/geroni/igaf122.3884

**Published:** 2025-12-31

**Authors:** Dan Andersen, Marla DeVries, Margaret Calkins, Christina Schilstra, Patrick Miller, Susan Ryan

**Affiliations:** Explore Digits, Sykesville, Maryland, United States; AgingIN, Linthicum Heights, Maryland, United States; IDEAS Institute, Cleveland Heights, Ohio, United States; Explore Digits, Rockville, Maryland, United States; Explore Digits, Rockville, Maryland, United States; Center for Innovation, Linthicum, Maryland, United States

## Abstract

The Green House (GH) model of nursing home care, characterized by fewer beds per unit, empowered staff, and a resident-directed philosophy, has long been associated with culture change innovation. To evaluate GH performance on national metrics of quality, we linked 53 sites that include GH homes to CMS data sources including the Five-Star Quality Rating System. We compared sites with GH homes to all other nursing homes across multiple domains: overall star rating, health inspections ratings, staffing ratings, quality measure ratings, and individual indicators such as case-mix adjusted staff time and quality measures. Sites with GH homes outperformed the national average across most metrics. For example, 78% of sites with GH homes received a 4- or 5-star rating for staffing compared to 31% nationally; sites with GH homes provided 56% more adjusted nurse aide time than the national average; and 68% of sites with GH homes earned a 4- or 5-star rating for quality measures compared to 49% of non-GH sites. Sites with GH homes also demonstrated fewer low-star ratings across every domain. Weekend staffing levels, a common area of decline in traditional homes, remained significantly higher in sites with GH homes. These differences are even more pronounced in a sub-analysis of GH-only campuses (i.e., sites with no traditional nursing home beds), though this subgroup was not reported separately due to small sample size. These findings suggest that adopting the GH model supports consistently higher quality outcomes, reinforcing its potential as a scalable strategy for improving nursing home care nationwide.

